# Who Provides Primary Care? An Assessment of HIV Patient and Provider Practices and Preferences

**DOI:** 10.4172/2155-6113.1000366

**Published:** 2014-10-27

**Authors:** Quen J Cheng, Elysia M Engelage, Tristan R Grogan, Judith S Currier, Risa M Hoffman

**Affiliations:** 1 UCLA Division of Infectious Diseases, Department of Medicine, David Geffen School of Medicine at UCLA, USA; 2 UCLA Division of Infectious Diseases and Center for Clinical AIDS Research and Education, Department of Medicine, David Geffen School of Medicine at UCLA, USA; 3 Department of Medicine Statistics Core, David Geffen School of Medicine at UCLA, USA

**Keywords:** HIV, AIDS, Primary care

## Abstract

**Background:**

Non-AIDS co-morbidities are emerging as the main health problems for those living with HIV, and primary care for this population is an evolving challenge. Recent studies have raised the question of whether specialists or generalists are best suited to provide HIV primary care, but patients’ actual usage patterns and the preferences of patients and providers have not been well studied.

**Methods:**

We anonymously surveyed 98 patients and eight HIV-specialized providers regarding primary care usage patterns and preferences at an academic HIV clinic in Los Angeles that serves insured patients.

**Results:**

Fifty-nine percent of patients use their HIV physician as their primary care provider, and 84% would prefer this model. Physicians were divided on their preferred role, with five out of eight desiring to provide both primary care and HIV care. All eight physicians rated their comfort with antiretroviral therapy and opportunistic infections greater than for non-AIDS co-morbidities. Eighty-one percent of patients and seven of eight providers were supportive of having a co-located primary care physician at the HIV clinic.

**Conclusions:**

We conclude that patients prefer integration of HIV and primary care, but providers have variable desire to serve as primary care physicians and may be uncomfortable with non-AIDS co-morbidities. This raises the need for improved patient-provider communication about primary care needs, and calls for novel ways of systematically providing primary care to HIV-infected patients.

## Introduction

Due to advancements in antiretroviral therapy (ART) HIV/AIDS has become a manageable chronic illness, and the life expectancy of HIV-infected patients has been extended to near-normal [1–3]. As patients with HIV live longer, they develop the same medical conditions that are common in an aging non-AIDS population but at higher rates [4]. Diseases such as hypertension, dyslipidemia, and diabetes, the “bread and butter” of general internal medicine and family medicine, are now the main health problems for most patients on ART in developed nations. Recent studies have raised the issue of whether these problems should be managed by generalists or HIV specialists. Fultz et al. demonstrated that general internists reported feeling significantly more comfortable treating these illnesses than infectious disease (ID) specialists [5], and Duffus et al. reported that ID specialists were four times more likely than other physicians to refer HIV-infected patients for hypertension and diabetes management [6]. Yet many patients with HIV receive primary care through specialist physicians, and the optimal system for managing the spectrum of non-AIDS problems is a matter of considerable debate [7]. Insurance coverage often dictates how people receive care, and historically, many individuals living with HIV have had limited access to non-AIDS care if covered by federal programs such as the Ryan White Program [8]. However, many HIV-infected patients do have access to primary care through private insurance, and this access is likely to increase with the implementation of the Affordable Care Act. The primary care usage patterns and preferences of insured HIV patients have not been widely studied.

The Center for AIDS Research and Education (CARE), part of the University of California, Los Angeles (UCLA) David Geffen School of Medicine, provides HIV care for approximately 1,000 patients in the greater Los Angeles area. CARE serves patients with health insurance and is staffed by seven infectious disease specialists, one internist who completed an HIV fellowship and one hematology/oncology physician who also serves as an HIV provider. The majority of patients are on ART with well-controlled, chronic HIV infection and high rates of non-AIDS co-morbidities. Although CARE patients have access to PCPs at sites distant from the clinic, whether they continue to see their PCP or rely on their HIV providers for general medical care is not known. It has also been unclear whether physicians at CARE consider themselves primary care providers to their patients. To answer these questions and investigate potential underlying determinants of primary care usage patterns and preferences, we anonymously surveyed both patients and providers at CARE.

## Materials and Methods

### Study design

Patients were included in the study if they had a diagnosis of HIV infection and had been receiving regular care at the clinic for greater than six months. We excluded patients being seen at CARE for research studies only. Providers were included if they were UCLA-employed physicians seeing patients at CARE. Surveys were performed over a four-month period from June 2012 to September 2012. The study was approved by the UCLA internal review board.

Two anonymous surveys were designed, one for patients and one for providers (supplemental file). The patient survey consisted of 13 questions. Patients were invited to participate in the survey at appointment check-in, and completed surveys were placed in a box at the front desk. Patients were asked to provide information about their age, sex, and which CARE physician was their HIV provider. Patients were then asked whether they see a PCP in addition to their HIV provider, and whether they consider their HIV provider to be their PCP. If they stated that they see an outside PCP, they were asked whether they did so only because they were required by insurance, and how many times a year they saw this PCP. Patients were then asked to select which non-AIDS medical problems they had out of a list of eight common diagnoses, how many medications they take daily in addition to their HIV medications, and how many specialist physicians they see yearly besides their HIV provider or PCP. Lastly, they were asked whether they would prefer to receive primary care services from their HIV provider or from a separate PCP, and if their HIV provider was not their PCP whether they would prefer to have a PCP located within the same clinic as their HIV provider.

The provider surveys consisted of nine questions and were collected anonymously via a designated folder in clinic. Providers were asked about their training background and years since completion of highest level of training. Providers were then asked to approximate the size of their patient panel and rate on a scale of one to five (one being “very uncomfortable” and five being “very comfortable”) their comfort level with HIV management including ART and opportunistic infections, and comfort level with each of the following medical topics: hypertension, hyperlipidemia, chronic obstructive pulmonary disease, diabetes mellitus, mood disorders such as depression and anxiety, liver disease, age-appropriate health maintenance, and chronic pain. They were asked to estimate using quartile ranges the percentage of patients in their practice for whom they serve as both HIV provider and PCP, and the percentage of their patients that actively see a PCP outside of CARE. They were also asked to estimate the percentage of their patients that see at least one additional specialist yearly. Lastly, they were asked whether they would ideally want to provide both HIV and primary care to their patients, as well as whether they would like to have PCPs co-located at CARE to provide primary care services.

### Data analysis

For quantitative variables the mean, median, standard deviation, and inter-quartile ranges were calculated using Microsoft Excel. For categorical variables frequency tables were obtained, and associations between categorical variables were assessed using Fisher’s exact test. If both variables were measured on an ordinal scale, the Spearman correlation was computed. A one-way ANOVA was performed to test for a difference in average age between the categorical variables of whether the HIV provider was the PCP. Statistical analyses were performed using IBM SPSS V22 (Armonk, NY). P-values<0.05 were considered statistically significant.

## Results

### Patient characteristics and healthcare preferences

A total of 101 patients agreed to participate in the study, and 98 surveys were completed for a completion rate of 97.0%. The median age of patient respondents was 48.5 years (IQR 40.3–53.0), and 97.0% (N=95) were male. Sixty percent (N=56) of patients identified at least one non-AIDS co-morbidity, and 86% (N=82) of respondents were taking at least one non-AIDS related medication with 27% (N=26) taking four or more of these medications daily. Seventy-one percent (N=67) of respondents reported seeing at least one other medical specialist annually in addition to their HIV provider. The most common co-morbidities were hypertension (27%), dyslipidemia (27%), and depression (26%).

Fifty-nine percent (N=57) of patients in our survey identified their HIV provider at CARE as their current PCP. Thirty-nine percent (N=37) stated that they see a PCP in addition to their HIV provider. Nineteen patients reported having appointments with their outside PCP less than once per year, and 23 patients reported they saw an outside PCP only because they were required to by their insurance company. Overall, 84% (N=80) of patients prefer their HIV provider be their PCP or already use their HIV provider as such. If patients were required to have a separate PCP, 65% (N=57) responded that they would prefer these two physicians to be located in the same clinic versus 19% (N=17) who would prefer separate clinic locations and 16% (N=14) who had no preference. A subset of respondents (32%) were patients of a physician who is an internist with HIV fellowship training. Subgroup analysis from this provider’s patients compared with patients from our specialist providers revealed no statistically significant differences in responses.

The likelihood of a patient using their HIV provider as their PCP was not associated with the patient’s age (p=0.79), the number of non-AIDS medical problems (p=0.28), the number of non-AIDS medications (p=0.23), or the number of other specialists seen per year (p=0.74) (Table 1). There was also no correlation between use of an HIV physician as PCP and which HIV provider a patient was seeing (p=0.37).

### Characteristics of physicians and their primary care perceptions and preferences

Eight out of nine CARE providers completed the survey for a completion rate of 89.0%. Four out of eight respondents had been practicing for over 15 years since completion of their highest level of training, and one of the eight had been practicing fewer than five years.

Four out of eight CARE physicians believe 75 –100% of their patients use them as their PCP, an overestimation of the actual 59% based on patient responses. Likewise, five out of eight providers believed that 0–25% of their patients see an outside PCP, an underestimation of the actual 39% who reported seeing an outside PCP. Five out of eight physicians responded that ideally they would want to provide both HIV and primary care services to their patients. Four were in favor of having primary care physicians co-located at CARE, while three had no preference and one preferred not to have additional physicians at CARE providing primary care services. Of the three who did not want to personally serve as PCP, two were in favor of PCP co-location and one had no preference. The one provider who preferred not to have PCP co-location also stated a desire to personally serve as PCP.

In the assessment of physician comfort level with HIV/AIDS, CARE providers responded with mean ratings of 4.9 and 4.6 for HIV management and opportunistic infections, respectively. All non-AIDS medical problems received comfort level ratings lower than 3.8, with the lowest scores being 2.6 for mood disorders and 2.8 for COPD (Table 2). All three of the providers who reported not wanting to provide primary care have been in practice greater than 15 years, and two of these three providers reported a mean comfort level of less than 3.0 for non-AIDS conditions. Among the five physicians who preferred to provide primary care for their patients the mean comfort level with non-AIDS co-morbidities was 3.5. Despite stating a preference for not providing primary care and a mean comfort of 2.3 with non-AIDS conditions, one respondent still estimated providing primary care to 75–100% of patients (Table 3).

## Discussion

Our study describes the primary care usage patterns and preferences of a panel of HIV-infected patients and their providers within an urban academic HIV clinic. A unique feature of our study is that the patients all had private insurance and therefore access to a generalist PCP. Despite this, we found that the majority of patients (59%) use their HIV physician for primary care, and an even greater majority (84%) would prefer their HIV physician provide both HIV and primary care. This finding was consistent across the entire population surveyed, regardless of patient age, specific HIV provider, or non-AIDS co-morbidities.

Although the patients strongly preferred integration of HIV and primary care, physician respondents were divided on whether they personally prefer to provide primary care. Notably, all three physicians who preferred not to serve as PCP had been in practice for over 15 years, suggesting that the difference in provider preference may be partly due to a cultural shift in HIV care. Perhaps younger physicians are choosing ID specifically for the primary care aspects of HIV medicine.

We also found important discrepancies between patient non-AIDS co-morbidities and their HIV providers’ comfort level in treating those diseases. Our findings are consistent with the published results of Fultz et al. [5] in which ID-specialists and internists practicing at HIV clinics were uniformly less comfortable than general internists with prescribing treatment for hypertension, hyperlipidemia, diabetes, and depression. In particular, Fultz et al. found that comfort level for treating depression was lower than for the other diseases in all three groups of physicians. HIV physicians’ discomfort with prescribing antidepressants was also described in a recent study by Bess et al. [9], and our study again reveals a striking discrepancy between the high prevalence of depression reported by patients and providers’ low comfort level with treating mood disorders.

Taken together, our results suggest a tension between patients’ desires for integrated care and HIV providers’ reservations about providing primary care. Interestingly, despite a self-reported discomfort with certain non-AIDS conditions as well as variable desire to personally provide primary care, physicians acknowledge that they do in fact serve as PCP, aligning with the patients’ preferred model of care. This creates potential mismatches in patient-provider preference, patient co-morbidity, and provider comfort level with non-AIDS illnesses.

The potential applications of these observations are wide-ranging. At the individual level, it calls attention to the critical importance of communication between patients and providers. Five percent of patients in our study did not know if their HIV provider was their PCP, suggesting a lack of overt dialogue about the spectrum of care to be provided. Lack of clarity could result in costly and time-consuming duplication of services, or important gaps in primary care. This study raises the need for direct communication between provider and patient about primary care, with clear expectations for where the patient will obtain this care. If both patient and physician agree that the HIV specialist will act as a PCP, our results would encourage a constant, careful assessment of the physician’s comfort level with non-AIDS medical conditions and low threshold for referral to specialists for specific areas self-identified as weaknesses. Encouraging specialists that act as PCP to maintain internal medicine proficiency and certification may be an additional effective strategy.

At a systems level, our study adds to the argument for more creative means of providing primary care to HIV-infected patients. One strategy is to bring primary care providers into the HIV clinic. These co-localized providers could be physicians trained in internal medicine or family medicine whose specific role is to see patients with complex non-AIDS co-morbidities as well as to provide basic primary care if preferred by the HIV provider. In our study this idea was well received by both patients and providers; only 19% of patients and one of eight providers were not in favor of the co-location model. Hiring non-physician staff specialists is another method to manage specific diseases. A recently described algorithm for the treatment of depression among HIV-infected patients features non-physician care managers who monitor antidepressant usage [10]. The idea of additional non-AIDS providers in the HIV clinic, whether physicians or otherwise, would begin to resemble a patient-centered medical home. The medical home has been studied in for other chronic diseases such as heart failure and diabetes with improvements in patient satisfaction and health outcomes [11,12], and could be an effective means of providing comprehensive HIV care.

Another promising strategy is to promote HIV training for general internists and thereby increase the number of providers who can adequately provide both HIV and primary care. This model has been successfully implemented in other developed nations such as Australia, where general practitioners complete a certification program in order to prescribe antiretrovirals [13]. A number of U.S. internal medicine residency programs are now offering HIV tracks for those interested in HIV primary care. One-year HIV fellowships after residency are also becoming commonplace, and the American Academy of HIV Medicine has a well-established HIV Specialist credentialing program. In this model, ID physicians may be used as consultants for more complex patients with resistance or opportunistic infections. This strategy may work well in more rural areas where there are fewer ID specialists.

Our study has a number of limitations. We intentionally surveyed a homogeneous population from a single center in order to understand the usage patterns and preferences of patients at our institution. Although we excluded patients who were only followed in research studies, CARE physicians conduct many clinical trials and the culture is strongly research-oriented, so this may limit the external validity of our study. Second, because this study was conducted at a single center where there are only nine practicing physicians, our provider sample size is small. Third, given the voluntary nature of the survey, our results are subject to response bias. Fourth, we randomly sampled patients presenting to our clinic over a four-month period, regardless of which provider they were seeing. The aggregate patient data may therefore be biased towards the practices of our highest-volume providers during that time. Finally, we did not collect information on the proportion of physician time spent in direct patient care. It is possible that physicians with limited clinical time were less interested in serving as PCPs.

In a population of insured patients with access to primary care providers, we found a strong patient preference for integrated ‘onestop’ HIV care and primary care. At the same time, we shed light on the challenges of meeting primary care needs with HIV specialists who may have a lower comfort level with common non-AIDS co-morbidities. Our study raises the need for clearer communication between patients and providers about primary care needs, and calls for novel strategies to systematically provide primary care to HIV-infected patients. The optimal way to meet both patient and provider goals and expectations for delivery of HIV primary care remains to be seen, and is a field that is ripe for additional pilot studies and comparative research to test different care models.

## Supplementary Material

Supplementary file

## Figures and Tables

**Table 1:** Primary care usage patterns. Fifty-nine percent of all patients identified their HIV provider as PCP. Age reported as mean (standard deviation). All other responses to categorical variables reported as frequency (percentage). There were no statistically significant associations between likelihood of a patient identifying their HIV provider as PCP and any of the surveyed variables.

	HIV provider is PCP	HIV provider is not PCP	Don’t know	p-value
Total respondents, n=98	57 (59%)	35 (36%)	5 (5%)	-
Age, n=98	47.5 (9.9)	46.1 (11.2)	46.0 (7.0)	0.79
Number of non-HIV medical problems, n=93				0.28
None	20 (54%)	16 (43%)	1 (3%)	
1	18 (57%)	10 (36%)	2 (7%)	
2	14 (78%)	3 (17%)	1 (6%)	
3	1 (20%)	4 (80%)	0 (0%)	
4 or more	2 (100%)	0 (0%)	0 (0%)	
Number of additional medications, n=95				0.23
None	7 (58%)	5 (42%)	0 (0%)	
1–2	11 (85%)	15 (43%)	4 (11%)	
2–3	11 (85%)	2 (15%)	0 (0%)	
3–4	7 (88%)	1 (13%)	0 (0%)	
Greater than 4	14 (54%)	11 (42%)	1 (4%)	
Number of additional specialists, n=94				0.74
None	13 (50%)	11 (42%)	2 (8%)	
1–2	32 (64%)	16 (32%)	2 (4%)	
2–3	3 (60%)	1 (20%)	1 (20%)	
3–4	4 (50%)	4 (50%)	0 (0%)	
Greater than 4	2 (50%)	2 (50%)	0 (0%)	

**Table 2:** HIV/AIDS and non-AIDS co-morbidities and provider comfort level. Number of patients reporting non-HIV medical co-morbidities are shown in column one, with percentage in parentheses. Providers’ mean comfort level with each disease, on scale of one to five with one being “very uncomfortable” and five being “very comfortable,” is shown in column two with standard deviation in parentheses.

	Number of patients (%)	Provider comfort level (standard deviation)
HIV Management	-	4.9 (0.35)
Opportunistic Infections	-	4.6 (0.52)
Diabetes	6 (6%)	3.1 (0.99)
Liver Disease	7 (8%)	3.3 (1.04)
COPD	1 (1%)	2.8 (0.46)
Hypertension	25 (27%)	3.8 (0.89)
Dyslipidemia	25 (27%)	3.8 (1.04)
Depression	24 (26%)	2.6 (0.74)
Chronic pain	10 (11%)	3.0 (1.51)

**Table 3:** Summary of physician responses. Eight physician providers completed the survey. On a Likert scale of 1 (very uncomfortable) to 5 (very comfortable) providers rated their own comfort level with HIV, opportunistic infections, and the following non-HIV conditions: hypertension, hyperlipidemia, COPD, diabetes, mood disorders, liver disease, healthcare maintenance, and pain management.

Provider	Comfort with HIV management	Comfort with opportunistic infections	Mean comfort with non-AIDS conditions	Percentage patients to whom they think they serve as PCP	Desire to provide primary care	Would like PCP co- located in HIV clinic
A	5	5	3.5	51–75%	Yes	Yes
B	5	5	3.3	26–50%	Yes	Yes
C	5	4	3.3	76–100%	Yes	No preference
D	4	4	3.3	76–100%	Yes	No preference
E	5	5	2.3	76–100%	No	Yes
F	5	5	2.5	0–25%	No	Yes
G	5	4	3.8	51–75%	No	No preference
H	5	5	3.3	76–100%	Yes	No
