# Supplementary material for: Who Provides Primary Care? An Assessment of HIV Patient and Provider Practices and Preferences
Source: J AIDS Clin Res. Author manuscript; Available in PMC 2015 Apr 24. (PMC4409003; doi:10.4172/2155-6113.1000366)
Supplement: Supplementary file [file NIHMS665564-supplement-Supplementary_file.pdf]

A Study to Investigate the Preferences of Patient Health Practices

Patient Questionnaire

Purpose: We are asking you questions about your health problems and where you seek health care in order to understand how to improve the care we deliver to you. We appreciate you taking the time to complete this survey. All information provided is confidential and will not be included in your chart, medical record or shared with your physician.

Definition: For the purpose of this study, **"primary care provider**" is the doctor you contact first with any health-related questions, manages some, if not all, of your chronic conditions and who provides health maintenance such as vaccines, cancer screenings, etc. Primary care providers may also be known as general Internists, Family Practice, and/or general practice doctors.

Please circle the most appropriate answer or fill in the blank as directed.

1. Age: ____

2. Sex:
   a. Male
   b. Female
3. Which doctor is your HIV provider:
   a. Dr. Debika Battacharya
   b. Dr. Margrit Carlson
   c. Dr. Emery Chang
   d. Dr. Judith Currier
   e. Dr. Jordan Lake
   f. Dr. Raphael Landovitz
   g. Dr. Matthew Leibowitz
   h. Dr. Ronald Mitsuyasu
   i. Dr. Ardis Moe

4. Do you see a primary care provider in addition to your HIV provider at CARE?
   a. Yes
   b. No
   c. I don't know

5. Is your HIV provider your primary care provider?
   a. Yes
   b. No
   c. I don't know
6. I have primary care doctor outside of the CARE clinic only because I am required to by insurance.

   a. Yes
   b. No
   c. This statement does not apply to me.

7. If you have a primary care doctor who is not also your HIV doctor, how many times a year do you see this person?
   a. Less than 1 time per year
   b. 1-2 times per year
   c. 3-4 times per year
   d. Greater than 4 times per year

8. Do you have any of the following medical problems (circle all that may apply):
   a. Diabetes Mellitus
   b. Liver disease
   c. Lung Disease
   d. High blood pressure
   e. High cholesterol
   f. Chronic Kidney Disease
   g. Depression
   h. Chronic pain
   i. I don't know

9. In addition to your HIV medications, how many different medications, including vitamins, do you take each day (circle one answer)?
   a. No additional medicine
   b. 1-2 additional medicines
   c. 2-3 additional medicines
   d. 3-4 additional medicines
   e. Greater than 4 additional medicines

10. Besides your HIV provider or primary care physician (if applicable), how many specialist physicians do you see per year? For example, a specialist physician would be any of the following: gastroenterologist, cardiologist, endocrinologist, dermatologist, psychiatrist, hepatologist (liver specialist), etc.
    a. No specialist doctors within the last year
    b. 1-2 specialist doctors within the last year
    c. 2-3 specialist doctors within the last year
    d. 3-4 specialist doctors within the last year
    e. More than 4 specialist doctors within the last year

11. Would you prefer to receive primary care services from your HIV provider? These services include urgent care appointments for non-HIV related matters, vaccine administration, cancer screening, non-HIV chronic disease management (such as diabetes, high blood pressure, depression), etc.

a. Yes, I would prefer that my HIV doctor provided all of my general health care.
b. No, I would prefer to receive general health care from an Internist or Family Medicine doctor and see my HIV provider only for HIV care.
c. I have no preference for who does my general health care.
d. My HIV provider already does all of my primary care services.

12. If your HIV provider was not your primary care provider, would you prefer to have a primary care provider within the same clinic to provide these services?
    a. Yes, I would like to have an Internist/Family Practitioner in the same office as my HIV doctor to provide primary care services
    b. No, I would prefer to have my HIV provider in one location and my primary care physician at a different clinic.
    c. It does not matter to me if my providers are at the same clinic or different locations.

13. If your HIV provider is your primary care provider, on a scale of 1-10, how satisfied are you with these services? (Please circle only one answer.)

1 2 3 4 5 6 7 8 9 10

(Very dissatisfied)< ---> (Very Satisfied)

Please feel free to share with us any thoughts you may have regarding the primary care services you receive at the CARE clinic.

Thank you for your participation!

A Study to Investigate the Primary Care Preferences of HIV-Infected Patients at the UCLA CARE Clinic

Provider Questionnaire

Purpose: Currently, HIV patients receive primary care services from providers of differing backgrounds with different levels of comfort in providing primary care services. Our project aims to assess both the patients' and providers' views towards these services at the UCLA Center for Clinical AIDS Research and Education (CARE). We would like to assess your view on certain primary care topics as well as your assessment of your patients' healthcare practices. We appreciate your participation in this survey.

Please circle the appropriate answer or fill in the blank as directed.

1. Training Background (Please circle one):
    a. Infectious Disease
    b. Internal Medicine
    c. HIV Specific Fellowship
    d. Family Practice
    e. HIV specific fellowship
    f. Other (please describe)_____

2. Number of HIV positive patients under your care:
    a. <50 patients
    b. 51-100 patients
    c. 101-150 patients
    d. >150 patients

3. Number of years after completion of highest level of training:
    a. <5 years
    b. 5-10 years
    c. 11-15 years
    d. >15 years

4. On a scale of 1-5, with 1 being "very uncomfortable" and 5 being "very comfortable," How comfortable are you with the following conditions (please circle only one answer):
    a. HIV management 1 2 3 4 5
    b. Opportunistic infection treatment and prevention 1 2 3 4 5
    c. Hypertension treatment 1 2 3 4 5
    d. Hyperlipidemia treatment 1 2 3 4 5
    e. COPD treatment 1 2 3 4 5
    f. Diabetes Mellitus treatment 1 2 3 4 5
    g. Mood disorders (Depression, anxiety, etc.) treatment 1 2 3 4 5

   h. Liver disease treatment 1 2 3 4 5
   i. Age appropriate healthcare maintenance (breast cancer screening, cervical cancer screening, bone densitometry, colorectal cancer screening, vaccine administration, etc.) 1 2 3 4 5
   j. Pain management 1 2 3 4 5

5. To what percentage of patients in your practice do you serve as both HIV provider and primary care provider?
   a. 0-25%
   b. 26-50%
   c. 51-75%
   d. 76-100%
   e. I don't know

6. What percentage of your patients has a primary care provider that they see outside of the CARE clinic?
   a. 0-25%
   b. 26-50%
   c. 51-75%
   d. 76-100%
   e. I don't know

7. What percentage of your patients sees at least one specialist yearly?
   a. 0-25%
   b. 26-50%
   c. 51-75%
   d. 76-100%
   e. I don't know

8. Ideally, would you want to personally provide both HIV and primary care services to your patients at CARE?
   a. Yes, I want to provide both HIV care and primary care to my patients.
   b. No, I want to provide only HIV care to my patients.

9. Would you like to have primary care physicians co-located at the CARE clinic to provide primary care services?
   a. Yes, I would like to have a primary care physician located at CARE to provide their services to patients.
   b. No, I prefer to have the CARE clinic focus on HIV services only.
   c. I have no preference as to having a primary care physician co-located at CARE.

10. We welcome any comments you may have regarding primary care services at the CARE clinic.

Thank you for your participation!
